# Pilot study of a pediatric metronomic 4-drug regimen

**DOI:** 10.18632/oncotarget.358

**Published:** 2011-12-05

**Authors:** Nicolas André, Sylvie Abed, Daniel Orbach, Corinne Armari Alla, Laetitia Padovani, Eddy Pasquier, Jean Claude Gentet, Arnauld Verschuur

**Affiliations:** ^1^ Service d'Hématologie et Oncologie Pédiatrique, Hôpital pour Enfants de La Timone, Marseille, France; ^2^ Metronomics Global Health Initiative, Marseille, France; ^3^ Service d'Oncologie Pédiatrique, Institut Curie, Paris, France; ^4^ Service d'Oncologie Pédiatrique, Grenoble, France; ^5^ Service de Radiothérapie, Hôpital de La Timone, Marseille, France; ^6^ Children's Cancer Institute Australia, Lowy Cancer Research Centre, University of New South Wales, Randwick, NSW, Australia

**Keywords:** Cmetronomic chemotherapy, pediatric oncology, angiogenesis, immune system

## Abstract

**Background:**

Metronomic chemotherapy (MC) is defined as the frequent administration of chemotherapy at doses below the maximal tolerated dose and with no prolonged drug-free break. MC is gaining interest as an alternative strategy to fight resistant cancer.

**Objective:**

to assess the safety of 4 drug MC regimen in pediatric patients with refractory or relapsing various tumors types.

**Setting:**

From November 2008 to December 2010, in three academic pediatric oncology centers, 16 children (median age 12 years old; range 5.5-20) were included in this pilot study. This treatment was proposed to children with refractory disease for whom no further effective treatments were available. Most frequent diagnosis were medulloblastoma/cerebral PNET (5) osteosarcoma (5), and one case each of nephroblastoma, high grade glioma, Hodgkin lymphoma, rhabdomyosarcoma, neuroblastoma and kidney rhabdoid tumour. The MC regimen consisted in cycles of 56 days (8 weeks) with weekly vinblastine 3 mg/m^2^ (week 1-7), daily cyclophosphamide 30 mg/m^2^ (days 1-21), and twice weekly methotrexate 10 mg/m^2^; (days 21-42), and daily celecoxib 100 mg to 400 mg twice daily (days1-56) followed by a 2-weeks chemotherapy break. Adverse events were determined through laboratory analysis and investigator observations.

**Results:**

One objective response was observed in a patient with Hodgkin lymphoma, and 4 patients experienced disease stabilization and continued their treatment for 3 cycles (24 weeks) or more. At last follow-up, 7 patients (43%) are alive including 1 still undergoing treatment. During the overall 36 cycles of treatments received by patients, 4 grade IV toxicities and 24 grade III toxicities were observed in 11 cycles in only 10 different patients.

**Conclusion:**

The metronomic regimen we report here was well tolerated and associated with disease stabilization. This regimen is currently being evaluated in a national multicenter phase II study.

## INTRODUCTION

Metronomic chemotherapy (MC) is defined as the frequent administration of chemotherapy drugs at doses below the single maximal tolerated dose and with no prolonged drug-free breaks [1]. MC has been reported to significantly reduce adverse events (AEs) usually associated with chemotherapy. Although clinical data of MC in pediatric oncology remains sparse [1, 2], this approach may be well suited and represents a genuine alternative solution for children with poor prognosis or refractory disease [3, 4], potentially as a maintenance therapy following multimodal treatment [5]. Furthermore, its low cost and limited toxicity make MC a very attractive therapeutic option in low- and middle-income countries [6, 7].

Here, we aimed at developing a new multidrug metronomic protocol for children, integrating the different mechanisms of action of MC. Indeed, although MC had initially been considered to be an anti-angiogenic therapy [8], recent findings have highlighted new effects, which all likely contribute to treatment efficacy [1]. These effects include the stimulation of the anticancer properties of the immune system, re-induction of tumor dormancy as well as potential direct effects against cancer cells [1, 9].

The potential of etoposide and temozolomide used in a metronomic fashion has already been reported [3, 4, 5, 10]. However, since potential mutagenic effects, including myelodysplasia and secondary leukemias, were reported with these agents when administered at more conventional dosing [11, 12], they were not included in this study. Instead, we designed a metronomic regimen relying mainly on oral medications and used a continuous low-dose methotrexate (MTX) / low-dose cyclophosphamide backbone published in 2002 by Colleoni *et al*. [13]. Lower doses of cyclophosphamide were used to avoid high hematologic toxicities since cyclophosphomide was combined with others anticancer agents. Vinblastine, which had already been included in metronomic protocols [14, 15], was also added to our protocol. This drug only adds little hematological toxicity at the dosage of 3mg/m^2^ and microtubule-targeting agents are known to be the most potent Anti-angiogenic chemotherapeutics so far [16, 17] and bring potential beneficial immunologic effect [18]. Lastly, celecoxib has been added as a 4^th^ agent in this metronomic combination. Celecoxib has been part of most pediatric metronomic regimen [3, 4, 5, 10, 14] as it adds potential anti-angiogenic effect and tumor sensitization to chemotherapy [19] and very limited toxicity. Here, we report of a pilot study of a 4 drug metronomic regimen in children with relapsing, refractory solid tumors.

## PATIENTS AND PROTOCOL

This pilot study evaluated the use of MC with cycles consisting of weekly intravenous vinblastine 3 mg/m^2^ (week 1 to 6 (later changed to 7 see below)), daily oral cyclophosphamide 30 mg/m^2^ (days 1-21), twice weekly oral MTX 15 mg/m^2^ (then lowered to 10 mg/m^2^ see below) (days 21-42), and twice daily oral celecoxib 100-400 mg (days 1-42), followed by a 2-week therapy break (celecoxib was then kept during the break see below). All families gave consent before enrollment of the patients in the study. This treatment was proposed to children, aged from 3 to 21 years old, with refractory or relapsing cancer following treatment protocols available in our institutions, or to patients who were not eligible for phase I or II trials. All patients with an uncontrolled concurrent illness, active infection or unable to swallow oral medication were excluded from the study. Adverse events (AEs) were determined through laboratory analyses and investigator observations and grade using the NCI-CTC 3.0 criteria. Treatment was terminated upon tumor progression, following physician's decision, according to parent's will or due to unacceptable toxicity. Tumor size was evaluated using bidimensional measurement. Disease status evaluated using WHO response criteria (progressive disease: 25% increase, stable disease: neither partial response nor PD criteria met, partial response: at least 50% decrease and complete response: disappearance of all known lesion(s)).

**Figure 1:**
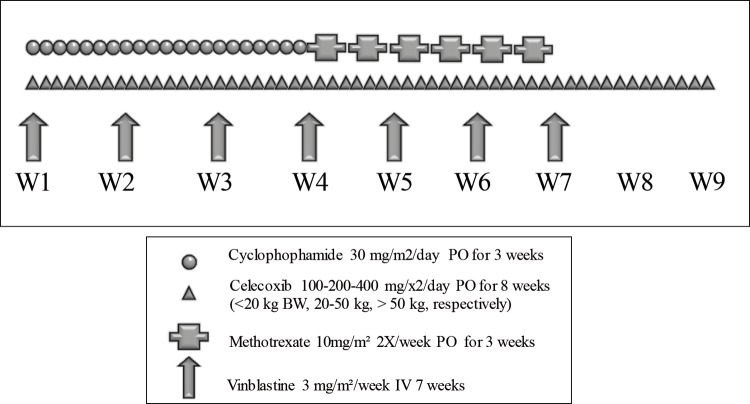
Metronomic 4 drugs regimen

From November 2008 to December 2010, 16 patients were included in this study, with a median age of 12 years old (5.5-20 years). There were 11 boys and 5 girls. Details of the study population and outcomes are reported in table 1.

**Table 1:** Patients characteristics and treatment outcomes

Pt nb	Sex	Tumour Type	Indication	Age (year)	Weight (kg)	Previous Lines of Treatment	HD-CT	RX	Previous Treatments Metronomic	Last TTP	Time on treatment	Best Response	Follow-up Weeks	Status
1	M	Medulloblastoma	PD	5.5	16	3	yes	yes	etoposide	8	8	PD	17	DOD
2	M	Rhabdoid Renal Tumor	PD	12	23	3	no	yes	etoposide	6	13	PD	33	DOD
3	M	Medulloblastoma	PD	9	22	3	no	yes	etoposide	8	14	PD	52	AWD
4	F	Osteosarcoma	M*	11.5	43	3	no	no	no	6	52	CR	68	AWD
5	M	Neuroblastoma	M	6	20	4	no	yes	COMBAT	6	8	PD	12	DOD
6	M	Nephroblastoma	M	12	46	4	yes	yes	no	5	14	PD	22	DOD
7	F	Osteosarcoma	M	16	40	3	no	no	no	25	6	PD	14	DOD
8	M	RMS	M**	20	54	2	no	yes	nvb-cyclo//tmz	16	52	CR	56	CR
9	F	Osteosarcoma	M***	16	52	2	no	no	no	6	24	CR	72	AWD
10	M	Hodgkin Lymphoma	PD	18	45	4	yes****	yes	no	?	20	PR	24	AWD
11	M	Glioblastoma	PD	9.5	44	3	no	yes	tmz	4	6	PD	6	DOD
12	F	Medulloblastoma	PD	12	34	3	no	yes	etoposide	6	6	PD	16	DOD
13	F	Supratentorial PNET	PD	11	30	2	yes	yes	no	34	14	PD	27	AWD
14	M	Medulloblastoma	PD	14	24	4	no	yes	no	32	6	PD	22	DOD
15	M	Osteosarcoma	PD	10	56	3	no	no	no	12	14	PD	30	DOD
16	M	Osteosarcoma	PD	8.5	27	1	no	no	no	84	24	PD	25	AWD

SD: Stable Disease; PD: Progressive Disease; AWD: Alive With Disease; DOD: Died Of Disease; M: Maintenance; Nvb: Navelbine; Tmz: Temozolomide; Cyclo: Cyclophosphamide; N.A: Non Available

°: this patient received zoledronic acid together with the metronomic regimen.

* this patient had a primary refractory metastatic osteosarcoma that progressed during the 2 first lines of chemotherapy. Metronomic chemotherapy was proposed as a maintenance therapy after obtaining surgical complete remission of both the primary tumour and bilateral lung metastasis. Monthly zoledronic acid was associated to the metronomic treatment.

** this patients had a third metastatic relapse of rhabdomyosarcoma. Metronomic maintenance was proposed after obtaining surgical CR and irradiation of the metastatic site.

*** this patient was proposed maintenance therapy after obtaining a 3rd surgical complete remission of a metastatic osteosarcoma.

**** this patient with Hodgkin lymphoma underwent both high dose chemotherapy followed by peripheral blood stem cell transplantation for a relapse and autologous bone marrow transplantation for a subsequent relapse.

¤ The COMBAT protocol is a combination of metronomic etoposide, temzolomide, celecoxib and retinoic acid.

¤¤ patients with Li Fraumeni syndrom who developed an osteosarcoma as a third tumor.

In children, who received the protocol as maintenance therapy and who were in CR at treatment initiation, the best status was considered as stable disease.

The treatment plan was slightly modified after treatment of the first 3 patients upon toxicity and clinical outcome. Thus, and following disease progression -suggesting active neo-angiogenesis during chemotherapy break-, it was decided to continue celecoxib administration between two cycles. A seventh vinblastine administration was also added on day 42. Then, as oral mucositis occurred during the second part of the cycle during which patients were taking oral methotrexate and celecoxib, methotrexate dosage was diminished from 15 mg/m^2^ to 10 mg/m^2^. Overall, 10 out of the 16 patients received the last implemented version of the regimen.

## RESULTS

At the end of the observation period, the mean duration of treatment was 17 +/− 14 weeks with 1 patient still on treatment. 7 out of 16 patients are alive with a mean follow-up of 28 +/− 15 weeks. Mean washout period was 5.6 +/− 7 weeks. The best response observed was one objective response in a child with Hodgkin disease who received a modified version of the protocol. Four disease stabilizations (25%) that lasted 24 weeks or more were also observed. All these patients received the implemented version of the regimen. Besides, rapid tumor progression was noted in 4 patients (25%) who did not complete the first cycle of treatment. When comparing time to progression (TTP) while receiving the experimental metronomic regimen with TTP during the previous line of treatment [20, 21], we found that TTP obtained with the experimental MC was longer in 8 patients (50%) (see table 1). However, overall there was no statistically significant difference between TTPs for respectively previous and current metronomic treatment (means 12.5 vs 17.1 weeks; Wilcoxon test, p=0.35).

Of particular interest, in 11 patients a decrease in pain and antalgic drug administration was rapidly observed after initiation of the metronomic treatment.

Most importantly, according to the National Cancer Institute Common Toxicity Criteria 3.0, with 24 grade-III and only 4 grade-IV AEs, tolerability was acceptable. They were mainly hematological (83% of toxicities and 75% of children). Six patients did not display any grade III or IV toxicities. It may be worthwhile to note that 4 of the patients (those with prior high dose chemotherapy followed by hematologic peripheral stem cell transplantation) accounted for 50% of all the AEs. No alopecia was reported and in all children except 1 who previously received high-dose chemotherapy, normal hair growth was noted. No grade III or IV nausea or vomiting was observed. Details are given in table 2. No patients stopped their treatment as a result of toxicity. Vinblastine dosage had to be reduced by 30% in 3 patients because of peripheral neurotoxicity (1 patient) and severe hematological toxicity (2 patients). Celecoxib was stopped in 2 patients due to renal insufficiency in one case and hemoptysis (related to lung metastasis) in the other, and MTX dosage was reduced in 4 patients because of grade II or III mucositis.

**Table 2:** Grade III/IV toxicities observed during the metronomic chemotherapy protocol

	Toxicity	Number of episodes	Number of patients
**Grade III**		24	10
	Absolute neutrophil count (>500-1000 G/L)	6	4
	Hemoglobin (6.5–8.0 g/dL)	4	3
	Leukocytes (1,000–2,000 G/L)	7	5
	Neuropathy	1	1
	Mucositis	1	1
	Renal Insuffisiency	1	1
	Platelets (10.000-50.000 G/L)	3	2
**Grade IV**		4	
	Absolute neutrophil count (>500-1000 G/L)	2	1
	Platelets < 10.000 G/L	2	2

## DISCUSSION

We report here the results of a 4-drug metronomic regimen in children with relapsing or refractory solid tumors. Of note, the treatment was also proposed to children at very high-risk of relapse, so that 3 patients received this treatment as a maintenance therapy (Table 1). Interestingly, 2 out of these 3 patients received the treatment for almost a year. For one patient the treatment is still ongoing. The second patient relapsed 2 months after stopping maintenance suggesting it may have been stopped to early as previously reported in a patient with medulloblastoma treated with the COMBAT regimen (22).

Treatment tolerance was similar to what has been reported with previous multi-drug metronomic regimens in pediatric populations [3, 4, 5, 10]. Mostly grade III/IV hematological toxicities were observed in 10 patients. It may be worthwhile to note that ¼ of the patients (those with prior high dose chemotherapy followed by peripheral hematopoietic stem cells transplantation) accounted for 50% of all the AEs. Six out of 16 patients (37.5%) did not display any grade III or IV toxicities. Therefore, this regimen is well tolerated in children and also allows treatment of previously very heavily treated who may not have been able to tolerate further MTD chemotherapy.

The initial combination of celecoxib and low dose MTX led to mucositis in 2 out of the 3 first treated patients. Although no pharmacokinetics study was performed to confirm the deleterious interaction between these two drugs, and despite the fact that cox-2 inhibitors did not change low-dose MTX pharmacokinetics in adults [23], it was decided to lower the doses of MTX. After the decrease in MTX dosage, the tolerance of the regimen was indeed improved and no further mucositis were observed.

One radiological response was noted after 2 cycles in a patient with Hodgkin lymphoma and 4 disease stabilizations were observed, according to classic WHO criteria. Although partial and complete responses have been reported in children receiving metronomic chemotherapy [3, 4, 5, 24], this type of therapy may more likely lead to tumor stabilizations [3, 4, 7, 10, 14]. In this regard it should be noted that the RECIST or WHO criteria may not be adapted in the evaluation of anti-angiogenic therapies [25]. Indeed, anti-angiogenic treatments are mainly considered as cytostatic, and as such are most likely to inhibit tumor progression rather than induce tumor regression. When considering the time to progression (TTP), which defines a clinical benefit by comparing the TTP while the patient is receiving the therapy immediately before the treatment of interest versus the TTP while under the experimental treatment, we found that the experimental MC regimen was associated with a longer TTP in 8 patients as compared to the previous lines of treatment. Such analysis of TTP paves the way for new clinical trial design to evaluate the efficacy of non-cytotoxic therapies such as metronomic treatments, which are less likely to produce tumor response assessed with RECIST.

Besides its potential effect in restraining tumor progression, MC seems to be able to help controlling pain in children with cancer. We previously reported fast improvement in pain control in children under MC [10] and Kivivuori et al. reported that MC could also help controlling pain symptoms in children with inoperable brainstem gliomas [26]. In the present study, we reported similar findings as 11 children (69%) experienced less pain, thus leading to a decrease in antalgic treatment, confirming the potential interest of MC in the palliative setting as already well established with for instance oral etoposide.

Among the 16 patients who were treated with this metronomic regimen, 7 had previously received MC, which mainly consisted in oral etoposide alone or combined with other metronomic agents. Interestingly, in these patients, TTP was equal or longer with the new metronomic protocol when compared to TTP with the previous metronomic regimen. Furthermore, patients for whom previous metronomic treatment helped controlling their disease also seemed to have their disease stabilized for longer period with the 4-drug regimen tested here, suggesting that cross resistance between metronomic regimens may not be systematic, opening the way for potential treatment with several lines of MC regimens and also re-challenge with the same treatment as recently reported by Sterba et al. [22].

Compliance is an important issue when considering oral treatments; we did not evaluate compliance in this study. Nevertheless, patients were always present for the planned dose of vinblastine injections suggesting a good adherence to treatment, although compliance with daily oral drugs and weekly injections are different type of constraints.

## CONCLUSION

The 4-drug regimen we report in this study was well tolerated. Although, efficacy cannot be fully assessed due to the small number of patients who were treated, the longer TTP observed when compared to previous lines of treatment strongly suggests a potential interest in children with refractory disease. A prospective national multicentric phase II study with ancillary biologic study is currently under way (clinicaltrial.gov - NCT01285817) as a result of this pilot study.
